# Green Solid Lipid Nanoparticles by Fatty Acid Coacervation: An Innovative Nasal Delivery Tool for Drugs Targeting Cerebrovascular and Neurological Diseases

**DOI:** 10.3390/pharmaceutics16081051

**Published:** 2024-08-08

**Authors:** Annalisa Bozza, Valentina Bordano, Arianna Marengo, Elisabetta Muntoni, Elisabetta Marini, Loretta Lazzarato, Chiara Dianzani, Chiara Monge, Arianna Carolina Rosa, Luigi Cangemi, Maria Carmen Valsania, Barbara Colitti, Ezio Camisassa, Luigi Battaglia

**Affiliations:** 1 Department of Drug Science and Technology, University of Turin, Via Pietro Giuria 9, 10125 Turin, Italy; valentina.bordano@unito.it (V.B.); arianna.marengo@unito.it (A.M.); elisabetta.muntoni@unito.it (E.M.); elisabetta.marini@unito.it (E.M.); loretta.lazzarato@unito.it (L.L.); chiara.dianzani@unito.it (C.D.); chiara.monge@unito.it (C.M.); ariannacarolina.rosa@unito.it (A.C.R.); luigi.cangemi@unito.it (L.C.); luigi.battaglia@unito.it (L.B.); 2 Department of Chemistry, University of Turin, Via Pietro Giuria 7, 10125 Torino, Italy; mariacarmen.valsania@unito.it; 3 Nanostructured Interfaces and Surfaces (NIS) Interdepartmental Centre, University of Turin, Via Quarello 15/a, 10135 Torino, Italy; 4 Department of Veterinary Sciences, University of Turin, Largo Paolo Braccini, 2, 10095 Grugliasco, Italy; barbara.colitti@unito.it; 5 Aulina s.r.l., Via Aulina, 14, 12035 Racconigi, Italy; info@aulina.it

**Keywords:** intranasal delivery, normal pressure hydrocephalus, neuroinflammation, antioxidant, green, solid lipid nanoparticles

## Abstract

Cerebrovascular and neurological diseases are characterized by neuroinflammation, which alters the neurovascular unit, whose interaction with the choroid plexus is critical for maintaining brain homeostasis and producing cerebrospinal fluid. Dysfunctions in such process can lead to conditions such as idiopathic normal pressure hydrocephalus, a common disease in older adults. Potential pharmacological treatments, based upon intranasal administration, are worthy of investigation because they might improve symptoms and avoid surgery by overcoming the blood–brain barrier and avoiding hepatic metabolism. Nasal lipid nanocarriers, such as solid lipid nanoparticles, may increase the nasal retention and permeation of drugs. To this aim, green solid lipid nanoparticles, obtained by coacervation from natural soaps, are promising vehicles due to their specific lipid matrix composition and the unsaponifiable fraction, endowed with antioxidant and anti-inflammatory properties, and thus suitable for restoring the neurovascular unit function. In this experimental work, such green solid lipid nanoparticles, fully characterized from a physico-chemical standpoint, were loaded with a drug combination suitable for reverting hydrocephalus symptoms, allowing us to obtain a non-toxic formulation, a reduction in the production of the cerebrospinal fluid in vitro, and a vasoprotective effect on an isolated vessel model. The pharmacokinetics and biodistribution of fluorescently labelled nanoparticles were also tested in animal models.

## 1. Introduction

Cerebrovascular diseases include a variety of conditions, such as ischemic and haemorrhagic stroke, vascular malformations, and vascular cognitive impairment, with an enormous impact in terms of mortality and morbidity. However, a common feature of such diseases is neuroinflammation [1,2], which can also actively participate in the development of neurological disorders [3]. Indeed, oxidative stress, with the consequent inflammatory reaction, induces an alteration in the neurovascular unit (NVU), which is formed by endothelial cells, pericytes, and astrocytes and plays a crucial role in protecting the brain and forming the blood–brain barrier (BBB). Moreover, the interaction between the NVU and the choroid plexus is critical for maintaining brain homeostasis, regulating cerebral blood flow, and producing cerebrospinal fluid (CSF) [2].

The disruption of normal CSF circulation and turnover is believed to contribute to the development of many cerebrovascular and neurological diseases [4]. CSF is produced by the four choroid plexuses inside the ventricular system of the brain. Here, there is the choroid plexus epithelium (CPE), consisting of a single layer of cuboidal to low cylindrical secretory cells residing on a basement. Together with the circum-ventricular organs and the arachnoid membrane, it constitutes the so-called blood–CSF barrier (BCSFB), stabilized by tight junctions at the luminal membrane [5]. Indeed, in healthy individuals, CSF is constantly produced to circulate through the brain and be subsequently reabsorbed into the bloodstream. Disturbances in CSF production and reabsorption underlie the physiopathology of hydrocephalus, a disease characterized by an accumulation of CSF and consequent dilatation of the brain ventricles [6].

Among its various forms, idiopathic normal pressure hydrocephalus (NPH) is a common neurological disease in the elderly, with a prevalence of 1 to 3%, characterized by peculiar clinical signs (known as the Hakim’s triad): cognitive impairment, gait disturbance, and urinary incontinence [7]. Despite that its physiopathology remains unclear [8,9], recent findings indicate a major role of venous failure in the brain capillaries [10]. Therefore, NPH might represent an example of the previously mentioned interconnection between neurological and cerebrovascular diseases.

Currently, a programmable-valve ventricular–peritoneal shunt is the gold-standard therapy for NPH, as it allows non-invasive CSF pressure adjustments to optimize clinical improvement. However, a pharmacological therapy would be needed, either as an adjuvant for improving symptom recovery or as an alternative to shunt surgery, in the case of contraindications [11]. Unfortunately, the poor availability of experimental models capable of truly recapitulating the human condition, as well as the lack of efficient brain delivery of specific drugs, has hampered such an approach so far.

Indeed, the main limitation for NPH-specific drugs would be the BBB, which prevents the passage of about 98% of the potential candidates from the blood to the brain [12]. Within this concern, intranasal administration would offer several practical advantages to the patients (non-invasive, painless, and well tolerated), while a rapid drug absorption and a quick onset of action may be obtained, avoiding the hepatic pre-systemic metabolism [13]. In fact, nose-to-brain delivery works due to the unique connection between the brain and the external environment provided by the olfactory bulb and trigeminal nerve in the nasal cavity.

However, since several factors can limit drug uptake by the nasal mucosa (including mucociliary clearance), biocompatible lipid nasal nanocarriers have been investigated, since they can enhance bioadhesion to the nasal mucosa and protect the encapsulated drug from biological degradation and efflux transporters. Liposomes showed a tendency to increase the brain uptake of encapsulated drugs, owing to their flexibility [14]. Nonetheless, some stability issues and technological problems affect their manufacturing. On the contrary, solid lipid nanoparticles (SLNs) can be manufactured at low costs, are able to encapsulate poorly water-soluble drugs, and show good physical–chemical stability [15].

Within this concern, fatty acid coacervation is a low-energy-consuming and solvent-free preparation method, which allows SLNs made-up of fatty acids to be obtained, starting from their alkaline soaps, following proton exchange [16]. Of note, natural soaps, obtained by the saponification of solid butters and fats, can be exploited as the raw material to obtain green SLNs by the fatty acid coacervation process [Italian Patent application 102022000021093]. Green SLNs might have innovative properties for the intranasal delivery of drugs targeting cerebrovascular and neurological diseases. Indeed, the unsaponifiable fraction, derived from the starting butters, is maintained in the SLNs and can confer antioxidant and vasoprotective properties, potentially suitable for restoring the NVU. Moreover, the presence of poly-unsaturated fatty acids (PUFAs) in the lipid matrix is beneficial for neurovascular health [17,18], whilst the mono-unsaturated ones (i.e., oleic acid) can promote permeation through the nasal mucosa [19,20]. Finally, such SLNs are stabilized by biocompatible surfactants, which favour the bioadhesion to the olfactory epithelium [15].

In this experimental work, green SLNs were formulated through fatty acid coacervation and fully characterized from a physico-chemical standpoint. Then, in vitro and ex vivo studies were carried out to assess their potential as drug vehicles for cerebrovascular/neurological diseases. Moreover, green SLNs were loaded with a combination of two drugs, suitable for reverting the NPH physiopathology, and tested on available cell models: bumetanide (Bume), which can decrease CSF production by blocking the NKCC1 symporter in the choroid plexuses [21,22,23,24], and dexamethasone (Dexa), which may reduce brain oedema that, in turn, increases liquid leakage from the bloodstream. Finally, green SLNs were labelled with two fluorescent probes (6-coumarin and rhodamine B), suitable for mimicking the behaviour of such drugs [25], and underwent pharmacokinetics and biodistribution studies in healthy rats after intranasal administration.

## 2. Materials and Methods

### 2.1. Chemicals

1-heptanesulfonic acid sodium salt, 500 kDa fluorescein isothiocyanate (FITC)-conjugated dextran, 6-cou, 60,000–90,000 molecular weight (MW) dextran, Bume, chloroform, *trans*-cinnamic acid, dimethyl sulfoxide (DMSO), ethanol, 9000–10,000 MW 80% hydrolysed polyvinyl alcohol (PVA 9000), linoleic acid, linolenic acid, porcine mucin, and rhodamine B (rhod B) were from Merck (St. Louis, MO, USA). Arachidic and behenic acid, hexane, and hydrochloric (HCl) and palmitic acid were from Fluka (Buchs, Switzerland). Sodium chloride was from Carlo Erba Reagents s.r.l. (Cornaredo, Italy). Glyceryl monopalmitate was from Serva (Tulsa, OK, USA). Acetonitrile MS grade, docusate sodium salt (AOT), methanol, N,O-bis(trimetilsilil)trifluoroacetamide (BSTFA), pyridine, formic acid, and oleic and stearic acid were from Merck (Darmstadt, Germany). Lactic acid, potassium hydroxide (KOH), and sodium hydroxide (NaOH) were from A.C.E.F. (Fiorenzuola d’Arda, Italy). Phosphoric acid was from VWR (Radnor, PA, USA). Dexa was from Alfa Aesar (Haverhill, MA, USA). Glyceryl monostearate was from Goldschidt (Essen, Germany). Glyceryl mono-oleate was from Evonik (Essen, Germany). The 10% (*v*/*v*) foetal bovine serum (FBS), 100 μg/mL penicillin–streptomycin–amphotericin, 2 mM L-glutamine, and high-glucose Dulbecco’s modified Eagle’s medium (DMEM) were from Aurogene Srl (Rome, Italy). Isoflurane was from Alcyon (Cherasco, Italy). Mango and Shea butters were provided by Aulina s.r.l. (Racconigi, Italy). Distilled water was purified using a MilliQ system (Millipore, Bedford, MO, USA). All solvents used (Sigma-Aldrich, St. Louis, MO, USA) and (CD_3_)_2_SO have analytical purity. All other chemicals were of analytical grade and used without further purification. Lupeol cinnamate was a kind gift from Prof. Taglialatela-Scafati’s lab [26].

### 2.2. Cells

Primary human umbilical vein endothelial cells (HUVECs) were isolated from human umbilical veins and maintained as previously described [27]. The use of HUVECs was approved by the Ethics Committee of the “Presidio Ospedaliero Martini” of Turin (protocol 263-07/NF and pr_SanPlac/16) and conducted in accordance with the Declaration of Helsinki. Primary choroid plexus epithelial cells (CPECs) were obtained from sheep at the slaughterhouse, right after regular slaughter, and cultured in DMEM (4500 mg/L) supplemented with 2 mM L-glutamine, 100 μg/mL penicillin–streptomycin–amphotericin, and 10% (*v*/*v*) FBS, at 37 °C in a humidified 5% CO_2_ atmosphere incubator.

### 2.3. Animals

Male Wistar rats (Charles River, MA, USA), weighing 250 g, were housed in standard facilities, handled, and maintained according to our Institutional Animal Care and Use Committee ethical regulations. Rats were kept under controlled environmental conditions (24 ± 1 °C, 50–60% humidity, 12 h light and dark cycles, lights on at 7:00 a.m.) and given ad libitum access to food and water. Aortas for isolated vessel experiments were explanted after animal sacrifice, according to a protocol approved by the Italian Ministry of Health (56105.N.ZMT/2018 and 56105.N.WSP/2023). Experiments on animal models were performed according to an experimental protocol approved by the University Bioethical Committee and the Italian Ministry of Health (Aut. N. 814/2018-PR).

### 2.4. Synthesis and Characterization of Conjugated Linoleic and α-Linolenic Acids

Conjugated fatty acids were prepared and characterized according to Miki Igarashi et al. [28], using 21% (*w*/*w*) KOH in ethylene glycol. The purity of the peak of the conjugated linolenic acid isomer was calculated by UHPLC-PDA at 240 nm and amounted to 4%. Since various conjugated linolenic fatty acids are formed during the synthesis, the isomer with a retention time of 45.4 min was selected for quantification, as described in Section 2.9.2.

### 2.5. Rhod B-AOT Ion Pair Formation and Characterization

An ion pair between the fluorescent probe rhod B and AOT was prepared to enhance its entrapment in the lipid matrix [29]. Briefly, rhod B was dissolved in water (8 mg/mL); then, a solution of AOT (4.5 mg/mL) was added, with a molar excess of AOT to rhod B of 2:1, to completely precipitate the 1:1 ion pair. Afterwards, the precipitate was centrifuged at 10,000 rpm for 10 min (Allegra^®^ 64 R centrifuge, Beckmann Coulter, Palo Alto, CA, USA) and washed twice with distilled water. To verify the ratio between rhod B and AOT, a certain amount of the ion pair was dissolved in methanol and read spectrophotometrically (rhod B: λ = 550 nm). The logarithmic coefficient of diffusion (logD) between water and chloroform of rhod B and the rhod B-AOT ion pair was determined as follows. A rhod B water solution (5 µg/mL) was prepared and divided into two samples. While the first one was kept as is, in the other one, 20 µL of AOT water solution (4.5 mg/mL) was added (molar ratio rhod B:AOT 1:2). In separate experiments, the two solutions were extracted with an equal amount of chloroform (1:1) in a separatory funnel, and the absorbance of the water phase was read spectrophotometrically (rhod B: λ = 550 nm) before and after the extraction. To determine the water solubility of rhod B and the rhod B-AOT ion pair, in separate experiments, 0.5 mg of the two compounds was added to 1 mL of water and stirred for 1 h at room temperature. Then, the suspensions were centrifuged at 14,000 rpm for 5 min (MPW55, Medical Instruments, San Lazzaro di Savena, Italy) to precipitate the insoluble portion, and the supernatant was diluted and read spectrophotometrically (rhod B: λ = 550 nm).

### 2.6. Saponification of Vegetal Butters and Fats

The saponification of Mango and Shea butters was performed by the cosmetic company Aulina s.r.l. Two distinct traditional non-continuous saponification methods were used for both Mango and Shea butters: hot saponification (max temperature reached by the soap paste = 98 °C) and cold saponification (max temperature reached by the soap paste = 35 °C). In both methods, vegetal fats were inserted into the reactor and melted at the specific temperature. The alkaline agent (sodium hydroxide) was then introduced under continuous stirring in an appropriate dilution and with 0.1% excess compared to the saponification indexes of the butters. Saponification times vary depending on the saponification method: 3 weeks for cold saponification and 12–15 h for hot saponification. The obtained natural soap paste underwent a maturing phase, which lasted from 2 days for the hot method up to 21 days for the cold one. During this period, the soap was maintained at 35% humidity and 23 °C.

### 2.7. SLN Formulation

Green SLNs were prepared by the fatty acid coacervation method [16]. Briefly, Mango and Shea soaps were dispersed in water, and the mixture was then heated to 70 °C under stirring (300 rpm) to obtain a clear micellar solution. The temperature was decreased to 50 °C; then, either lactic or phosphoric acid 1M, mixed with a 10% PVA 9000 solution, was added dropwise as the coacervating solution, until complete fatty acid precipitation. Finally, the obtained suspension was cooled under stirring. For fluorescently labelled SLNs, 6-cou, rhod B, and the rhod B-AOT ion pair, pre-dissolved in a small amount of DMSO (10 mg/mL), were added to the SLNs after acidification up to a final concentration of 0.05 mg/mL. The drug combination of Bume and Dexa was loaded in SLNs by dissolving the compounds in a small amount of ethanol (25 and 12.5 mg/mL) and adding it to the micellar solution, up to a final concentration of 1 mg/mL and 0.5 mg/mL, respectively.

### 2.8. SLN Characterization

#### 2.8.1. Size, Morphology, Differential Scanning Calorimetry (DSC), and Nuclear Magnetic Resonance (NMR)

The mean particle sizes (intended as the hydrodynamic diameter) and polydispersity of lipid nanocarriers were determined after 1 h and 30 days of storage at 4 °C, using the dynamic light scattering technique (DLS; 90 Plus, Brookhaven, NY, USA). Size measurements were obtained at an angle of 90° at a temperature of 25 °C. The homogeneity of the suspension was checked with optical microscopy (DM2500, Leica Microsystems, Wetzlar, Germany). Transmission Electron Microscopy (TEM) was performed with a S9000G FIB-SEM (Tescan, Brno, Czech Republic) after 2% osmium tetroxide staining. Microanalysis was performed by high-resolution Field-Emission Scanning Electron Microscopy (FE-SEM) 300 kV (Jeol Italia, Milan, Italy) with samples previously metallized with a 5 nm thick gold layer.

DSC was performed with a DSC 7 (Perkin-Elmer, Waltham, MA, USA). SLN pellets, obtained by ultracentrifugation at 26,000 rpm (Allegra^®^ 64 R centrifuge, Beckmann Coulter, Palo Alto, CA, USA), as well as free fatty acid mixtures, obtained from natural soaps by proton exchange with phosphoric acid in the absence of stabilizers, were freeze-dried (Modulyo Freeze Dryer, Edwards Alto Vuoto, Trezzano sul Naviglio, Italy) before being analysed. Palmitic and stearic acid were used as references. The samples to be characterized, consisting of 0.5–1 mg freeze-dried product, were placed in conventional aluminium pans and heated from 30 °C to 80 °C at 2 °C min^−1^. The degree of crystallinity of SLNs was estimated as the ratio between the melting enthalpy/g lipid of SLN pellet and that of the corresponding fatty acid mixtures, using Pyris Version 3.71 data processing software.

The ^1^H-NMR spectra of Mango and Shea fatty acids were recorded on a Jeol ECZ-R 600 spectrometer (Jeol Italia, Milan, Italy) at 600 MHz. The NMR analysis was performed to confirm the ratio between the different fatty acids obtained by gas chromatography coupled to mass spectrometry, as described in Section 2.9.1. Indeed, the ratio between total -CH_2_, CH=CH, and CH_3_ was considered. Samples were prepared as described above for DSC analysis and dissolved in (CD_3_)_2_SO.

#### 2.8.2. Drug and Fluorescent Probe % Recovery and Entrapment Efficiency

The % recovery, defined as the ratio between actual and theoretical compound concentration in SLN suspension, and % entrapment efficiency (EE%), defined as the ratio between lipid entrapped and total compound in suspension, were determined after lipid matrix separation by ultracentrifugation. To this aim, 0.5 mL of SLN suspension was diluted with 0.5 mL of a 30% water solution of 60,000–90,000 MW dextran and centrifuged at 26,000 rpm for 15 min (Allegra^®^ 64 R centrifuge, Beckmann Coulter, Palo Alto, CA, USA) to layer the lipid pellet above the supernatant. The % recovery of the compounds loaded in SLNs was calculated as the ratio between the sum of the amounts recovered in the supernatant and in the pellet vs. the total weight, while the EE% was the ratio between the amount in the pellet vs. the sum of the amounts in the supernatant and in the pellet. In the case of the fluorescent probes, the supernatant (unentrapped probe) was diluted 10-fold in methanol and centrifuged. The entrapped probe was extracted from the lipid pellet with 0.75 mL methanol, following dilution in 0.25 mL water and centrifugation, to discard the precipitated lipids. Obtained samples were analysed spectrophotometrically (6-cou: λ = 450 nm; rhod B: λ = 550 nm). In the case of drugs, the supernatant (unentrapped drugs) was diluted 1:4 in methanol. The entrapped drugs were extracted from the lipid pellet with 0.2 mL methanol, following dilution in 0.1 mL water and centrifugation, to discard the precipitated lipids. Obtained samples were analysed by HPLC, as described below.

#### 2.8.3. PDA-HPLC

HPLC analysis was performed with a YL9110 Quaternary Pump, equipped with a YL 9160 diode array detector (PDA-Yang Lin, Anyang, Republic of Korea), linked to Clarity software v3.0.4.444 for data analysis (Yang Lin, Anyang, Republic of Korea). The column was C18 Mediterranean Sea 5 μm 15 × 0.46 (Teknokroma, Barcelona, Spain). The eluent was constituted by phosphate buffer 0.05 M, pH 5.8, and methanol as the mobile phase A and B, eluted with a gradient analysis (min 0: 92% A, 8% B; min 10: 35% A, 65% B; min 15: 35% A, 65% B; min 17: 92% A, 8% B; min 20: 92% A, 8% B). The flow rate was set at 1 mL/min. The PDA was set at λ = 254 nm and λ = 241 nm for Bume and Dexa, respectively. The retention time was 12 and 15 min for Bume and Dexa, respectively.

### 2.9. Lipid Matrix and Unsaponifiable Fraction Characterization

The lipid matrix of the SLNs was isolated from the outer phase as follows: 500 μL of SLNs was mixed with 500 μL of distilled water, and 20 mg of sodium chloride was added to each sample to improve the phase separation between the pellet (lipid matrix) and the supernatant. Samples were then centrifuged at 26,000 rpm for 15 min. The pellet was isolated, while the supernatant was placed in a wide-necked amber vial, and both were freeze-dried (Modulyo Freeze Dryer, Edwards Alto Vuoto, Trezzano sul Naviglio, Italy).

#### 2.9.1. Gas Chromatography Coupled to Mass Spectrometry (GC-MS)

GC-MS was used to quali-quantitatively characterize the lipid composition of the SLN pellet. The SLN blank and the Mango and Shea soaps were qualitatively analysed as well, for comparison. To this aim, the freeze-dried samples underwent derivatization with BSTFA to obtain the trimethylsilyl derivatives. Specifically, 80 μL of pyridine and 120 μL of BSTFA were added to approximately 4 mg of dried pellets/blank/soap, the solution was then heated to 60 °C for 30 min in a water bath. The samples obtained were subsequently analysed by GC-MS (Agilent 6890 GC unit coupled to an Agilent 5973 MSD, Agilent, Little Falls, DE, USA). The separation was performed on a column of 5% phenyl polydimethylsiloxane (30 m, d*_c_* 0.25 mm, d*_f_* 0.25 μm). Helium was used as the carrier gas; the flow rate was set at 1 mL/min in constant flow, and the injector temperature was 250 °C. The detector temperature (MS) was 280 °C; the electron impact ionization was set to 70 eV; and the mass range was 50–800 *m*/*z*. The oven temperature programme was as follows: hold 1 min at 50 °C; increase to 300 °C at 3° C min^−1^; and hold 10 min at 300 °C. The compounds were identified by comparing their mass spectra and retention indices with those reported in the literature and in the available databases (Wiley and Nist) and confirmed by co-injection of the commercial reference standards (fatty acids and residual monoglycerides). The quantification was carried out with the external standard calibration method in selected ion monitoring (SIM) mode, selecting a target ion for each compound (Table S1). Each compound was quantified using the calibration curve generated with the corresponding reference standard. Data were processed using GCMS Solution v4.30 software (Shimadzu, Tokyo, Japan).

#### 2.9.2. Ultra-High-Performance Liquid Chromatography Coupled to Photodiode Array Detector and Mass Spectrometry (UHPLC-PDA-MS)

The components of the unsaponifiable fraction were extracted from Mango and Shea soaps and from the corresponding SLN pellet and supernatant, as follows. In total, 1 mL of methanol was added to the freeze-dried samples (50 mg of Mango and Shea soaps, ~4 mg of SLN pellet, and ~30 mg of SLN supernatant were extracted) that were subjected to ultrasound extraction for 10 min. The supernatant was collected, and the operation was repeated. The 2 mL of methanolic extract was combined, dried under a nitrogen steam, and resuspended in 200 μL of methanol which was filtered with a 0.20 μm hydrophobic polytetrafluoroethylene (PTFE) filter (CPS Analitica, Milano, Italy) and analysed by UHPLC-PDA-MS. The SLN blank (extraction without matrix) was extracted using the same method and analysed for comparison. The analyses were performed on a Shimadzu Nexera×2 system equipped with an SPD-M20A photodiode detector in series with a triple quadrupole Shimadzu LCMS-8040 system provided with an electrospray ionization (ESI) source (Shimadzu, Tokyo, Japan), as described by Marengo A. et al., 2017 [30], without any modification except for the gradient program (5% B for 3 min, 5–15% B for 17 min, 15–25% B for 10 min, 25–75% B for 12 min, 75–100% B for 10 min, and 100% B for 30 min for a total pre-running and post-running time of 93 min). The chromatograms were integrated at 270 nm. Semiquantification in Mango and Shea soaps was performed using the single-point calibration method. For cinnamic acid (peak 1), its commercial standard of reference was used at a concentration of 100 μg/mL. Conjugated fatty acid derivatives (peak 2) were quantified with the synthesized conjugated linolenic acid as a reference compound at a concentration of 500 μg/mL. Lupeol cinnamate, synthesized by the Taglialatela-Scafati group, at a concentration of 500 μg/mL, was used for the quantification of lupeol cinnamate (peak 3) and its derivatives (peaks 4, 5, and 6). The ratio between the compounds in the pellet and in the total suspension was calculated using the following equation: PA_p_/(PA_p_ + PA_s_), where PA_p_ is the peak area of the compound in the pellet, and PA_s_ is the peak area of the compound in the supernatant.

### 2.10. Release Studies

#### 2.10.1. Release Studies of Fluorescent Probes

The release of fluorescent probes from labelled Mango and Shea SLNs was assessed through the dialysis bag (14 KDa) method at room temperature. In total, 2 mL of labelled SLNs was put in the bag and dialysed vs. 200 mL normal saline under magnetic stirring. In separate experiments, a release study with rhod B-AOT-loaded formulations was also carried out in the presence of porcine mucin to evaluate the effect of the mucosal components on the ion pair displacement after intranasal administration. To this aim, 1 mL of porcine mucin (10 mg/mL) was added to the dialysis bag. Free fluorescent probes were used as controls to assess the dialysis membrane permeability. They were prepared as follows: rhod B was dissolved in distilled water; 6-cou suspension was obtained by preparing a stock solution in DMSO (10 mg/mL) and then diluting it in distilled water. The quantification of the released probes was carried out at scheduled times by the withdrawal of 3 mL of the receiving phase, which was analysed spectrophotometrically (6-cou: λ = 450 nm; rhod B: λ = 550 nm).

#### 2.10.2. Release Studies of Drugs

The release of Bume-Dexa from Shea SLNs was assessed through the dialysis bag (14 KDa) method at room temperature. In total, 3 mL of loaded SLNs was put in the bag and dialysed vs. 30 mL of phosphate buffer 0.2 M pH 7.4 under magnetic stirring. The quantification of the released drugs was carried out at scheduled times by the withdrawal of 100 µL of the receiving phase, which was analysed by HPLC (see Section 2.8.3).

### 2.11. Cell Studies

#### 2.11.1. Cell Viability Assay

Cell viability was determined on HUVECs and on sheep CPECs by a standard MTT [tetrazolium salt (3-(4,5-dimethylthiazol-2-yl)-2,5-diphenyltetrazolium bromide)] assay, performed after 72 and 24 h treatment, respectively [31].

#### 2.11.2. CSF Secretion

CPECs (5 × 10^4^ cells/well) were plated on Transwell^®^ tissue culture inserts (3.0 µm polyester membrane, 12 mm, Corning Incorporated, ME, USA) on 12-well plates previously coated with mouse laminin (5 µg/mL, 2 h, Sigma-Aldrich, St. Louis, MO, USA) and grown at 37 °C. The medium was replaced every 2–3 days. To verify the presence of the cells’ impermeable monolayer, the barrier integrity was evaluated through transepithelial electrical resistance (TEER, Millicell ERS-2, Merck-Millipore, MA, USA). Solute permeability was assessed using 500 kDa fluorescein isothiocyanate (FITC)-conjugated dextran (0.11 mg/mL). At day 10, once the presence of monolayers had been verified, studies were performed: a known amount of FITC–dextran was added to both apical and basolateral chambers, and, at scheduled times (0, 60, 120, 180, 240, and 360 min), 20 µL was collected from the apical chamber. Fluorescence was measured at λ_exc_ = 485 nm and λ_em_ = 535 nm using a VICTOR X4 (PerkinElmer, MA, USA) plate reader. CSF flow (µL/cm^2^) was calculated based upon variations in FITC-conjugated dextran concentration, according to the literature [32,33,34]. Untreated cells were compared to free and SLN-loaded drug combinations of Bume and Dexa. Free drugs dissolved in DMSO (15 mg/mL and 7.5 mg/mL, respectively) were diluted to 1:1500 in medium; drug-loaded SLNs, prepared as described above, were diluted to 1:100 in medium. In both apical and basolateral chambers, the final concentration of Bume and Dexa was 0.1 µg/mL and 0.05 µg/mL, respectively.

### 2.12. Vasoprotection in Rat Aorta with Endothelium Impairment Induced by Pyrogallol

The experiments were performed on rat thoracic aortic rings, prepared as previously described [35]. Briefly, male Wistar rats weighing 180–200 g were anaesthetized with isoflurane and killed by cervical dislocation. The aorta was immediately removed, dissected free of fat and connective tissue, and cut into rings approximately 3–4 mm wide. Endothelium-intact rings were mounted under 1.0 g tension in organ baths containing 30 mL Krebs bicarbonate buffer, maintained at 37 °C and gassed with 95% O_2_-5% CO_2_ (pH 7.4). The rings were allowed to equilibrate for 60 min, and endothelium integrity was assessed with 10 μM acetylcholine (ACh) in rings precontracted with 1 μM phenylephrine. A ≥75% relaxation of phenylephrine-induced tone was considered a sign of a functional endothelium. The effect of Shea SLNs or catechin, taken as a reference, on acetylcholine-induced relaxation in aortic rings pre-incubated with pyrogallol was studied, as published elsewhere [36]. The aortic rings with a functional endothelium were randomly divided into four groups: (1) control: endothelium-intact rings incubated in Krebs solution; (2) Shea SLNs: endothelium-intact rings incubated with 0.3 mL of SLNs 4% (1:100 dilution in buffer) or reference catechin (10 mM) for 30 min; (3) pyrogallol: endothelium-intact rings incubated with 500 μM pyrogallol for 15 min; and (4) Shea SLNs plus pyrogallol: endothelium-intact rings incubated with 0.3 mL of SLNs 4% (1:100 dilution in buffer) or the reference catechin (10 mM) for 30 min and 500 μM pyrogallol for the last 15 min. After the incubation time, the rings were washed, 1 μM phenylephrine was added to the organ baths, and then acetylcholine (10^−9^–10^−5^ M) was added cumulatively. Responses were recorded by an isometric transducer (1 g resting tension) connected to the MacLab System PowerLab (ADInstruments Ltd., Oxfordshire, UK). Tension was measured and calculated as the percentage of contraction in response to phenylephrine (1 μM). Experiments were performed for *n* = 3–7.

### 2.13. Animal Studies

#### 2.13.1. Pharmacokinetics after Intranasal Administration

In total, 100 μL of the fluorescently labelled formulations under study was administered to rats, as described by Muntoni E et al., 2022 [25]. At scheduled times (0, 5, 15, 30, 60, 120, 180, and 360 min) after administration, blood samples were collected, and plasma was isolated and subjected to deproteinization [25]. The obtained supernatants were dried in a Speed vacuum (Hetovac, VR-I, De Mari Strumenti, Milan, Italy), reconstituted with 50 μL of 75/25 methanol/water solution and analysed by means of HPLC, as described below. Tests were carried out for *n* = 4 of each formulation under study.

#### 2.13.2. Biodistribution after Intranasal Administration

The administration of labelled formulations was performed as in the pharmacokinetic study. After animal sacrifice, performed 1 h after administration, plasma was withdrawn, and the organs (liver, spleen, kidneys, lungs, heart, and brain) were removed surgically. Blood samples were collected in heparinized tubes and centrifuged to isolate plasma. The freshly removed brain underwent capillary depletion to isolate brain capillaries from parenchyma [36]. The other organs were homogenized with UltraTurrax^®^ (IKA, Staufen, Germany) [25]. Tissue homogenates and plasma underwent deproteinization [25], while supernatants were dried in a Speed vacuum (Hetovac, VR-I, De Mari Strumenti, Milan, Italy), reconstituted with 50 μL of 75/25 methanol/water solution and analysed by means of HPLC, as described below. Experiments were performed for *n =* 4 of each experimental condition.

#### 2.13.3. HPLC–Fluorimetry

HPLC analysis was performed with a YL9110 Quaternary Pump, equipped with a Shimadzu RF-20 fluorescence detector (Shimadzu, Tokyo, Japan), linked to Clarity software for data analysis (Yang Lin, Anyang, Republic of Korea). 6-Cou and rhod B were analysed, as described by Muntoni E et al., 2022 [25].

### 2.14. Statistical Analysis

Results are reported as the mean ± SEM, and statistical analysis was performed using the Student’s *t* test for unpaired data and one-way or two-way ANOVA followed by Bonferroni’s Multiple Comparison Test (Prism Graphpad 5.0, Graphpad Software, San Diego, CA, USA, 2016).

## 3. Results

### 3.1. SLN Characterization

#### 3.1.1. Size, Morphology, and DSC

The composition and physico-chemical characterization of green SLNs is shown in Table 1. Noteworthily, SLNs showed no surface charge, since they are sterically stabilized by neutral polymer PVA 9000. The mean diameters of SLNs, determined by DLS, are in the 170–400 nm range, while the polydispersity is lower than 0.3. The SLNs mean size varies owing to drug loading and to the saponification method used. Of note, cold saponification and the loading of compounds negatively affects the particles’ mean size. Therefore, hot saponification was selected as the default method for the following studies. Noteworthily, during storage at 4 °C (Table S2), SLNs loaded with fluorescent probes maintained their size, while unloaded SLNs and drug-loaded SLNs increased in dimension, yet always showing a polydispersity lower than 0.3.

DSC thermograms are shown in Figure S1. Preliminarily, DSC analyses were performed with (1) the main saturated fatty acids (palmitic, stearic, and arachidic) that constitute the green SLNs and (2) the mixtures of fatty acids obtained by proton exchange (coacervation) from Mango and Shea soaps, in the absence of stabilizers (Figure S1A). As can be seen, stearic acid obtained by coacervation was found in the B crystalline form, which melts at 55 °C. The mixtures of fatty acids derived from Mango and Shea soap by proton exchange melt at 46 °C and 54 °C, respectively. Furthermore, they are characterized by a lower melting enthalpy, compared to pure saturated fatty acids (Table 2), resulting in broader peaks (Figure S1A). These phenomena may be due to the presence of liquid fatty acids (oleic, linoleic, etc.) in the mixture, which leads to a more amorphous structure, compared to the pure saturated fatty acids. Figure S1B shows the DSC of Mango and Shea SLNs. As can be noticed, the SLNs’ melting enthalpies match with those of the corresponding free fatty acid mixtures, while a slight decrease was noted for the melting temperature (Table 2). Such a melting point decrease can be due to the reduced particle size of the colloidal system, with a high surface-to-volume ratio [37], as well as the presence of impurities, surfactants, and stabilizers [16]. However, the solid state of the particles at room temperature was assessed and supercooled melts excluded [38].

**Figure 1:**
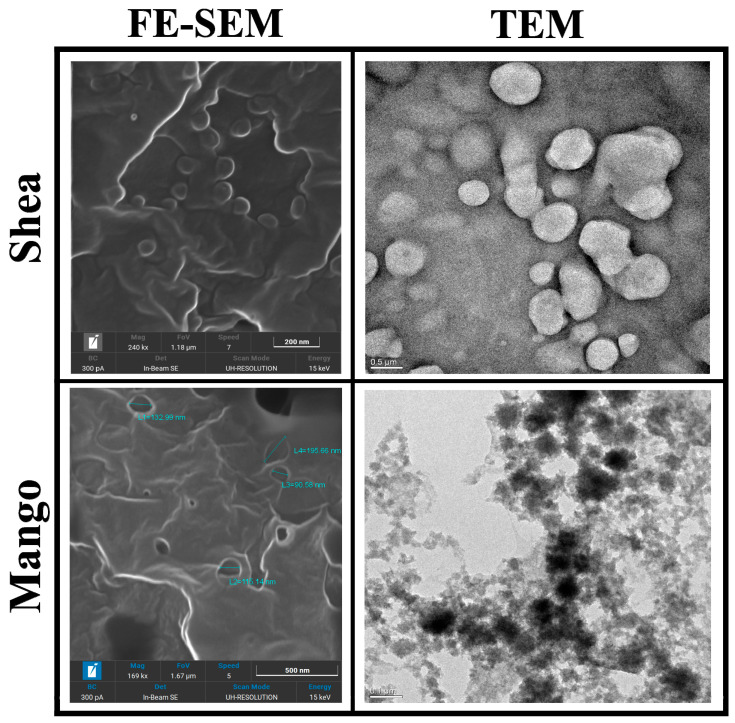
Transmission Electronic Microscopy (TEM) and Field-Emission Scanning Electronic Microscopy (FE-SEM) images of Shea and Mango solid lipid nanoparticles (SLNs). Markers used: 200 nm for Shea SLNs by FE-SEM, 500 nm for Shea SLNs by TEM, 500 nm for Mango SLNs by FE-SEM, and 100 nm for Mango SLNs by TEM.

Mango and Shea SLNs were preliminarily observed with optical microscopy (OM) in order to exclude the presence of aggregates or microparticles (Figure S2). In the case of labelled nanoparticles, a dotted signal was detected in correspondence to SLNs by fluorescence OM, thus confirming the probe’s entrapment into the lipid matrix (Figure S3). Moreover, Mango and Shea SLNs were characterized by TEM and FE-SEM. Mango SLNs were dark-contrasted (Figure 1), while Shea SLNs (Figure 1) showed a black staining only on the surface of particles. This is probably due to their different composition, which affects osmium tetroxide absorption onto nanoparticles. However, from FE-SEM microanalysis (Figure 1), in both cases, osmium tetroxide (evidenced with a white signal) can be noted on the SLNs’ surface. TEM and FE-SEM allowed us to appreciate the spherical shape and smooth surface of green SLNs.

#### 3.1.2. Drug and Fluorescent Probe Loading

In separate experiments, the selected drug combination (Bume and Dexa) and the fluorescent probes were loaded within green SLNs. In the case of the drug combination, lactic acid was used to precipitate SLNs, since it improves the stability of the suspension in the presence of the loaded compounds. The drug EE% was highly influenced by the lipid matrix employed; however, Bume was loaded with a higher EE% (74–83%) compared to Dexa (25–53%) (Table 1). In separate experiments, two fluorescent probes (6-cou and rhod B) were loaded in green SLNs to mimic the in vivo behaviour of potential drug candidates with different solubility. Indeed, rhod B, unlike 6-cou, is slightly water-soluble. Since this concern might result in a low EE%, rhod B-AOT was loaded in SLNs: in fact, the ion pair is less water-soluble (118.16 vs. 203.78 µg/mL) and more lipophilic (logD 0.99 vs. 0.75) than the free probe. Of note, in this way, an EE% higher than 85% was obtained for both the fluorescent probes; instead, the EE% of the free rhod B was between 38 and 55% (Table 1).

### 3.2. Lipid Matrix and Unsaponifiable Fraction Characterization

#### 3.2.1. Gas Chromatography Coupled to Mass Spectrometry (GC-MS)

The composition of the lipid matrix is shown in Table 3, and the GC-MS profiles of the derivatized samples are shown in Figure S4. Quantitative results were obtained using the external standard calibration method of the derivatized standard compounds in GC-MS-SIM, as described in Section 2.9.1. Solid fatty acids, such as palmitic, stearic, and arachidic acid, as well as liquid ones, such as oleic and linoleic acid, were detected. Also, residual monoglycerides, such as glyceryl stearate, glyceryl palmitate, and glyceryl oleate, were present, indicating that the complete saponification of the butters was not obtained by using either the cold or the hot method. However, residual monoglycerides are present to a greater extent with the cold saponification and are not involved in the fatty acid coacervation process, so they are likely responsible for the instability of the derived SLNs. On the other side, the quantity of linoleic acid, a ω-6 PUFA with reported anti-inflammatory properties [17,18], in the lipid matrix is also related to the saponification method, whereas cold processing grantees a higher stability for this component.

#### 3.2.2. NMR

Chemical shifts (δ) are given in parts per million (ppm). The following abbreviations are used to designate multiplicities: br. s = broad singlet, t = triplet, q = quintet, and m = multiplet.

^1^H-NMR Shea fatty acids ((CD_3_)_2_SO), 0.85 (t, CH_2_-CH_3_), 1.23 (m, CH_2_), 1.47 (q, CH_2_-CH_2_-COOH), 1.95–2.05 (m, CH_2_-CH=CH), 2.14–2.20 (t, CH_2_-CH_2_-COOH), 2.73 (t, CH=CH-CH_2_-CH=CH), 3.38 (br. s, COOH/OH), 3.83–4.05 (CH_2_-OH), and 5.28–5.35 (m, CH=CH/-CH-OCO).
^1^H-NMR Mango fatty acids ((CD_3_)_2_SO), 0.85 (t, CH_2_-CH_3_), 1.23 (m, CH_2_), 1.47 (q, CH_2_-CH_2_-COOH), 1.95–2.04 (m, CH_2_-CH=CH), 2.14–2.20 (t, CH_2_-CH_2_-COOH), 2.73 (t, CH=CH-CH_2_-CH=CH), 4.06 (br. s, COOH/OH), 3.83–4.05 (m, CH_2_-OH), and 5.28–5.35 (m, CH=CH/-CH-OCO).

Such values confirm the chemical composition of the fatty acid mixtures obtained after the proton exchange of Shea and Mango soaps.

#### 3.2.3. Ultra-High-Performance Liquid Chromatography Coupled to Photodiode Array Detector and Mass Spectrometry (UHPLC-PDA-MS)

The aromatic components of the unsaponifiable fraction and the semiquantification are reported, together with the ratio between the peak areas of the compounds in the pellet compared to their sum in the pellet and supernatant (Table 4). The corresponding UHPLC-PDA profiles (λ = 270 nm) are shown in Figure S5. Among the detected compounds, *trans*-cinnamic acid and lupeol cinnamate were confirmed by co-injection with the purified standard; the others were putatively identified based on the literature data. Besides the unsaponifiable compounds, the other major compounds detected by UHPLC-PDA-MS are conjugated fatty acids, putatively identified by the synthesis of conjugated linolenic fatty acid, reported in Section 2.4. They are probably formed from the free fatty acids present in the soap due to the alkaline conditions used for the saponification process. Unfortunately, it is not possible to determine the correct identity of these molecules, but this class of compounds is known in the literature for its anti-inflammatory properties [17,41,42,43,44,45]. Moreover, *trans*-cinnamic acid may be likely associated with the antioxidant properties of green SLNs [46]. Owing to an incomplete saponification, numerous triterpene cinnamyl ester derivatives were also present; among them, lupeol cinnamate has been identified, with important anti-inflammatory activities [47,48]. The unsaponifiable composition is similar between Mango and Shea SLNs, with some variations from a quantitative standpoint. The most polar compound (*trans*-cinnamic acid) is present mainly in the supernatant, while the conjugated fatty acids and the triterpene cinnamyl ester derivatives are more abundant in the pellet.

### 3.3. Release Studies

**Figure 2:**
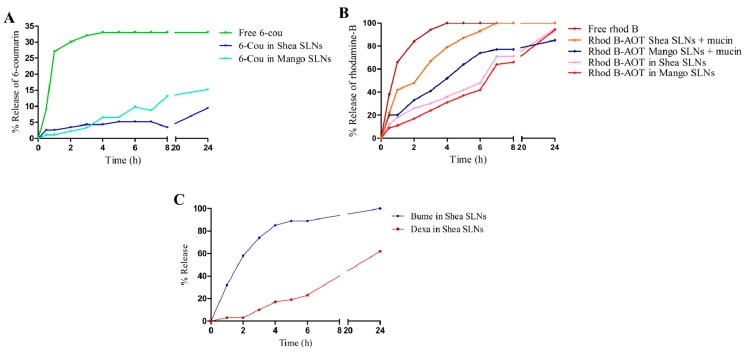
(**A**) % release of free 6-cou and 6-cou in Mango and Shea SLNs. (**B**) % release of free rhod B and rhod B-AOT ion pair from Mango and Shea SLNs, in the absence and in the presence of mucin. (**C**) % release of Bume-Dexa from Shea SLNs. Abbreviations: 6-cou: 6-coumarin; AOT: sodium docusate; rhod B: rhodamine B; SLNs: solid lipid nanoparticles.

The release of the two fluorescent probes from green SLNs to normal saline is shown in Figure 2A,B, while the release of the loaded drugs from Shea SLNs to phosphate buffer 0.2 M pH 7.4 is shown in Figure 2C. Consistent with their different solubility, a relevant difference between the two drugs can be observed at physiological pH. Indeed, since Dexa is lipophilic and almost water-insoluble, the release of the drug over time is modest. Despite that Bume is also lipophilic, a prompt release is obtained with 100% reached after 24 h. This happens because Bume sodium salt, which is formed at physiological pH, is slightly water-soluble. Regarding the fluorescent probes, experiments could not be performed in a larger amount of releasing medium due to the detection limit. Under such conditions, free 6-cou is even more insoluble in water than Dexa, and therefore, the release might not occur in a “sink condition”. Indeed, the release of the free 6-cou stops at 33%, likely due to the saturation of the acceptor buffer. Nevertheless, 6-cou in the green SLNs is integral in the lipid matrix, and its release is slower than that of the free probe. On the other side, the complete release of rhod B from SLNs is obtained at 24 h, with a similar pattern to Bume. Moreover, the addition of mucin, which binds hydrophilic compounds [52] and is capable of displacing the rhod B-AOT ion pair, slightly changes the probe release trend from SLNs. Given the conditions in which this study was carried out, it might be hypothesized that the release of the compounds from the lipid matrix occurred mainly by diffusion. Indeed, at room temperature, there is no melting of the lipid matrix.

### 3.4. Cell Studies

#### 3.4.1. Cell Viability Assay

**Figure 3:**
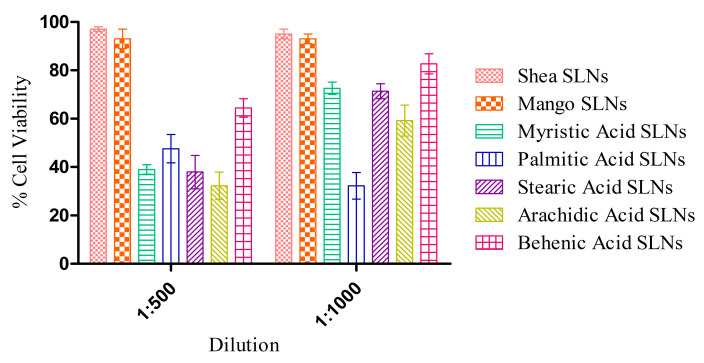
Cytotoxicity assay on HUVECs after 72 h exposure to Shea and Mango solid lipid nanoparticles (SLNs), compared to SLNs made up of synthetic soaps.

MTT experiments with HUVECs at 72 h (Figure 3) showed a negligible cytotoxicity for green SLNs, in comparison with coacervation SLNs previously obtained with pure synthetic soaps [16]. Indeed, the unsaponifiable fraction could have a role in the protection against the inflammatory response induced by the free fatty acids [53].

#### 3.4.2. CSF Secretion

**Figure 4:**
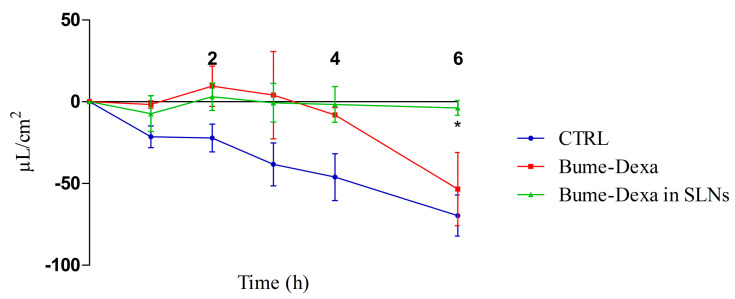
Sheep CPEC fluid secretion assay reported as µL/cm^2^ in the apical chamber. *n* = 3. Bume-Dexa in SLNs vs. CTRL at 6 h. Statistical analysis: two-way ANOVA, * *p* < 0.05. Abbreviations: Bume: bumetanide; CPECs: choroid plexuses endothelial cells; CTRL: control; Dexa: dexamethasone; SLNs: solid lipid nanoparticles.

In Figure 4, it can be observed that drugs, either free or loaded SLNs, decrease the production of CSF in a CPEC model. However, significant differences can only be observed between the untreated controls and the Bume-Dexa-loaded Shea SLNs at 6 h. Of note, the MTT assay for free and Bume-Dexa-loaded Shea SLNs showed a negligible cytotoxicity on CPECs of sheep origin.

### 3.5. Ex Vivo Studies on Isolated Vessels

**Figure 5:**
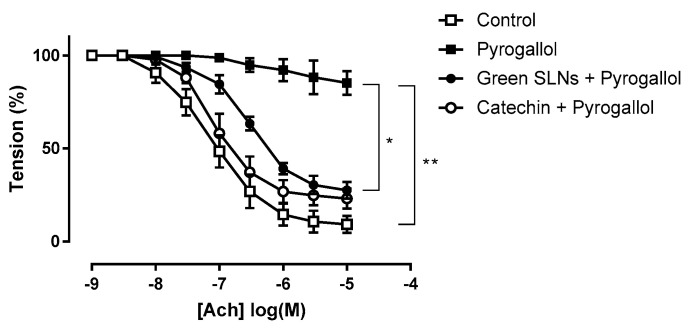
Ex vivo assay on isolated vessels showing the effect of green SLNs on acetylcholine-induced vasorelaxation in endothelium-intact rat aortic rings. Statistical analyses were performed using Student’s *t* test for unpaired data. ** *p* < 0.01 and * *p* < 0.05 vs. the pyrogallol group. Abbreviations: Ach: acetylcholine; PE: phenylephrine; SLNs: solid lipid nanoparticles.

Green SLNs, by virtue of the lipid matrix composition and the unsaponifiable fraction, were tested in an ex vivo model of endothelial dysfunction. 4% Shea SLNs were used in order to increase the concentration of the unsaponifiable fraction. Superoxide anion (O_2_^−^) generated by pyrogallol significantly reduced the maximum Ach-induced relaxation in aortic rings from 91 ± 2% to 15 ± 3% (*p* = 0.0051, *t* test). Treatment with catechin (10 mM), a well-known O_2_^−^ scavenger [54], restored Ach-induced relaxation to control levels (77 ± 3%; Figure 5). Treatment with 4% Shea SLNs without exposure to pyrogallol had no effect on the maximum relaxation, confirming that green SLNs alone did not impair endothelium function. Pre-incubation with 4% Shea SLNs attenuated the relaxation dysfunction induced by pyrogallol exposure, with a significant increase in the Ach-induced maximal relaxation to 72 ± 2% compared to 15 ± 3% in the pyrogallol group (*p* = 0.034, *t* test; Figure 5).

### 3.6. Animal Studies

**Figure 6:**
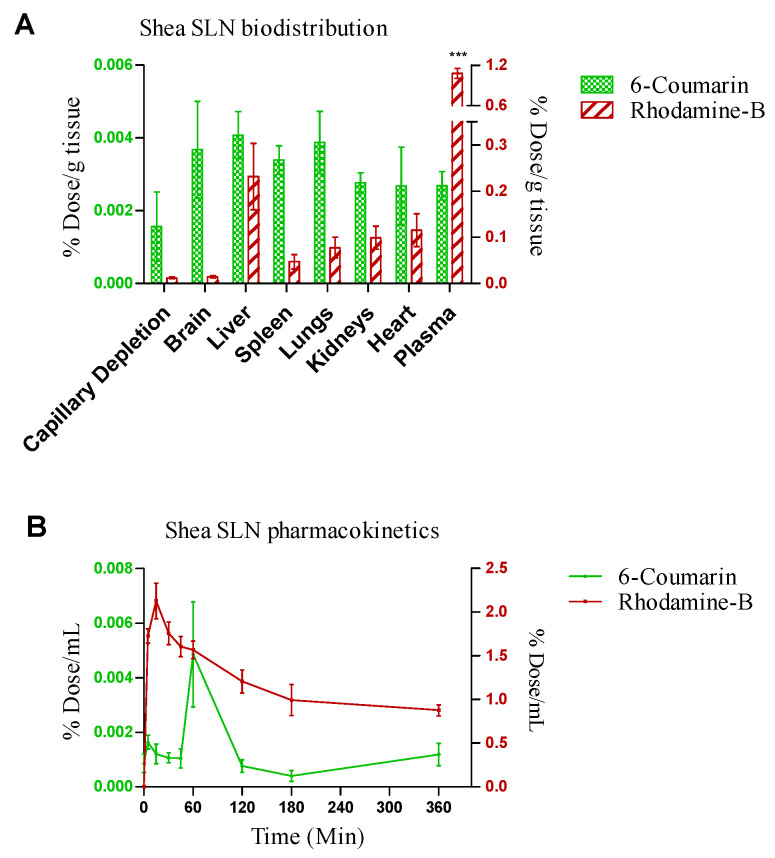
(**A**) Biodistribution and (**B**) pharmacokinetics of 6-coumarin- and rhodamine B-labelled Shea solid lipid nanoparticles (SLNs) in healthy Wistar rats after intranasal administration. Statistical analysis of (**A**) rhodamine B in plasma vs. rhodamine B in RES organs and brain: one-way ANOVA, *** *p* < 0.0001. (**B**) Rhodamine B- vs. 6-coumarin-labelled Shea SLNs: two-way ANOVA, *** *p* < 0.0001.

Shea SLNs labelled with 6-cou and rhod B-AOT were administered by the intranasal route to healthy rats. In pharmacokinetic studies, rhod B uptake into the bloodstream was nearly 100-fold higher compared to 6-cou. Moreover, rhod B uptake was rapid (T_max_ = 10 min); in contrast, the pharmacokinetic T_max_ of 6-cou was 1 h (Figure 6A). Biodistribution studies, shown in Figure 6B, were performed by sacrificing the animals 1 h after administration. In accordance with pharmacokinetic studies, a nearly 100-fold higher tissue accumulation was measured for rhod B compared to 6-cou. However, 6-cou distribution into the brain was proportionally more pronounced compared to rhod B. Moreover, plasma was the main site of accumulation for rhod B, with significant differences compared to the reticulo-endothelial system (RES) organs and brain.

## 4. Discussion

### 4.1. Physico-Chemical Standpoint

SLNs by coacervation are an established vehicle for drug and gene delivery, obtained by a solvent-free and low-energy-consuming technique [19,20,25,55,56,57,58,59]. However, pure synthetic fatty acids have been used so far. On the contrary, a natural soap has a complex composition (Table 3 and Table 4), which makes it difficult to obtain a homogeneous suspension by coacervation. Indeed, a mixture of three solid (palmitic, stearic, arachidic) and two liquid (oleic, linoleic) fatty acids is present in the lipid matrix. Moreover, some fatty acids are converted in their conjugated form during the saponification process. Finally, in the case of our solid butters, conventional saponification methods fail to reach complete ester hydrolysis. Indeed, residual monoglycerides are still present in the soaps, as well as cinnamates (triterpene alcohols cinnamates) in the unsaponifiable fraction. The residual monoglycerides represent a relevant hurdle for the stability of the nanoparticulate system obtained by coacervation. Of note, in this experimental work, by optimizing the operating conditions, spherical-shaped and controlled-sized green SLNs were obtained by coacervation from Mango and Shea soaps, which proved suitable for multiple drug and fluorescent probe delivery [Italian Patent application 102022000021093].

### 4.2. Composition–Activity Relationship

Noteworthily, synthetic fatty acid SLNs, except in the case of behenic acid, are activators of tumour necrosis factor alpha (TNF-α) [53]. This might contribute to a certain toxicity towards HUVECs after long-time exposure (72 h). On the contrary, green SLNs’ anti-inflammatory and antioxidant properties were demonstrated in vitro and ex vivo, since they were capable of overcoming the cytotoxicity of the free fatty acids (Figure 3) and protecting the isolated vessels from the damage induced by pyrogallol (Figure 5). Indeed, *trans*-cinnamic acid, one of the major components of Shea and Mango unsaponifiable fractions, can inhibit TNF-α-induced tissue factor expression in vascular endothelial cells, owing to B Cell Nuclear Factor Kappa-light-chain-enhancer (NF-κB) activation [60]. Moreover, it might act as a scavenger for the ROS produced by pyrogallol in the isolated vessel, increasing superoxide dismutase (SOD) and glutathione (GSH) levels in the hippocampus and cortex and being beneficial for neuroinflammation treatment by attenuating oxidative stress [46] and inhibiting lipid peroxidation [61]. Also, lupeol cinnamate present in Shea and Mango unsaponifiable fractions is well known for its strong anti-inflammatory properties [47] due to TNF-α, nitric oxide (NO), interleukin (IL), -1β (IL-1β), and -12 (IL-12) inhibition [48]. Indeed, lupeol cinnamate may decrease the expression of pro-inflammatory enzymes, such as inducible nitric oxide synthase (iNOS) and cyclooxygenase-2 (COX)-2 [47,48].

Finally, specific components of the green SLNs’ lipid matrix, that is, fatty acids and their conjugated derivatives, might be endowed with anti-inflammatory activity [17,41,42,43,44,45]. Indeed, LA plays a crucial role in the formation of various biologically active compounds through its self-oxidation [62]. The major compounds are 9S- or 13S-hydroxyoctadecadienoic acid (HODE) [17]. Both 9-HODE and 13-HODE are involved in modulating inflammation in the vascular wall. For instance, 13-HODE has been associated with increased cyclic adenosine monophosphate, which contributes to platelet/endothelial adhesion reduction [63], and HODEs may stimulate prostacyclin production in endothelial cells [62,64]. In addition, they act as agonists for peroxisome proliferator-activated receptor alpha (PPAR-α), which plays a key role in various cellular processes, including lipid metabolism and inflammation regulation [65,66]. Moreover, conjugated linolenic acid (cLNA) and derivatives strongly inhibit cyclooxygenase-1 (COX)-1 [45].

### 4.3. Pharmacological Standpoint

The aforementioned evidence makes green SLNs suitable for restoring NVU function, which is altered in the case of cerebrovascular and neurological diseases. Since NPH is an illustrative case of the interconnection between such diseases, green SLNs were loaded with a drug combination suitable for restoring hydrocephalus-injured brain. Such a combination included Dexa, acting on brain oedema, and Bume, capable of reducing CSF secretion by inhibiting the NKCC1 co-transporter, expressed in the CPE luminal membrane [67]. The effect of drug-loaded SLNs on CSF was investigated by using a specific secretion assay, performed in confluent Transwell, seeded with sheep primary CPECs. In serum containing medium, filter-grown monolayers displayed TEER values in the range of 100 to 150 Ω·cm^2^, in good correspondence to rat choroid plexus cells in vitro [68] and cell polarity. Moreover, they were not permeable to FITC–dextran (<2%) for up to 6 h. However, in such conditions, CPECs did not significantly express the choroid plexus-specific outward fluid secretion or active transport. Indeed, with 10% serum in the culture medium, an inward secretion (from the apical chamber towards the basolateral) was present, confirming the absence of active transport by the Na^+^/K^+^-ATPase transporter and the Na^+^/H^+^-antiporter [34,69], which selectively highlights the action of NKCC1 co-transporter [5], which is specifically targeted by Bume. Uniquely in the CPECs, the NKCC1 transport is outwardly directed due to the high intracellular ion concentrations, thus contributing to CSF production [67]. However, in CPECs cultured with 10% serum, such a transmembrane ion gradient might be lost, and thus, the NKCC1 transport turns inward, like in most of the tissues (i.e., the kidneys). Of note, such a mechanism was confirmed by flow inhibition obtained with the drug combination, which is even more evident when it is loaded in SLNs. On the contrary, active transport could be obtained on the serum-free grown monolayer (TEER range of 1500 to 2500 Ω·cm^2^) by using an incubation buffer containing 122 mM NaC1, 1mM CaC1_2_, 1 mM MgCl_2_, 4 mM KC1, 15 mM NaHCO_3_, 15 mM HEPES, 0.5 mM Na_2_HPO_4_, 0.5 mM NaH_2_PO_4_, 17.5 mM glucose, and 5 µg/mL insulin, pH 7.3 (Figure S6). However, the aim of this study was to mimic a pathological permeability dysfunction of CP, which can only be obtained by using serum in the culture medium [69].

### 4.4. In Vivo Fate

Nose-to-brain administration would be suitable for drug delivery in the case of the potential pharmacological treatment of NPH, due to its claimed advantages. Within this concern, green SLNs are a versatile platform, suitable for restoring the altered NVU and capable of delivering the drug combination systemically in the bloodstream and locally in the brain. Capillary depletion was carried out to verify the labelled SLN targeting the brain vessels that constitute the NVU, allowing us to appreciate the presence of both probes, especially 6-cou. Due to their size, likely only a small amount of green SLNs can reach the brain intact. However, in a previous study [70], it was reported that nanoparticles > 500 nm can also reach the middle and brain stem regions of the trigeminal nerve. Green SLNs are stabilized by bioadhesive PVA, suitable for slowing the nasal mucociliary clearance, and they contain oleic acid, which may act as a permeation enhancer on nasal mucosa, decreasing the impact of the size on clearance. Since in vitro release studies showed a comparable trend between Dexa and 6-cou, as well as between Bume and rhod B-AOT, these two fluorescent probes have been used to approximate the behaviour of such drugs in animal studies. Indeed, in vivo studies with nasally administered SLNs demonstrated a different fate for the loaded fluorescent probes, owing to their water solubility. 6-cou, which is integral to the lipid matrix, is associated with a delayed permeation and a poor systemic bioavailability, likely due to the relatively large size of green SLNs [70], which are excluded from fast transcellular/paracellular permeation. On the contrary, it could be hypothesized that the slow intraneuronal route plays a role in the nose-to-brain uptake of such nanocarriers, which is size-limited only by the average diameter of olfactory axons (100–700 nm) [15,25]. Rhod B, instead, is promptly released from SLNs, also thanks to mucin, which is present on the surface of the nasal cavity. Being a small MW molecule, released rhod B permeates easily and rapidly by the paracellular/transcellular route, with good systemic bioavailability but with low selectivity for the brain. Moreover, the high level of rhod B detected in plasma demonstrates a systemic-driven brain delivery, which is mediated by preliminary uptake by the lungs and the bloodstream [12]. Generally, the direct nose-to-brain uptake of compounds is desirable for cerebrovascular and neurological diseases; nonetheless, systemic uptake would also be beneficial in specific cases. Noteworthily, in the case of NPH, the systemic uptake of Bume may cause an increased elimination of fluids by the kidneys, thus reducing the blood pressure and, in turn, liquid extravasation from blood to the CSF [24].

## 5. Conclusions

In this experimental work, the operating conditions were optimized to obtain green SLNs by coacervation from natural Mango and Shea soaps, which were fully characterized from a physico-chemical standpoint. Owing to their specific composition, green SLNs showed promising vasoprotective activity in vitro and ex vivo, suitable for restoring the NVU, which is altered in cerebrovascular and neurological diseases. Given that NPH is a representative case of the interconnection between such brain diseases, green SLNs were loaded with a drug combination suitable for counteracting NPH physiopathology and tested in a fluid assay. Preliminary in vivo studies with nasally administered labelled SLNs showed that the uptake of loaded compounds depends upon their release at the nasal mucosa, whereas both systemic and brain uptake might contribute to the therapeutic efficacy. However, further in vivo studies on animal models, capable of recapitulating the human NPH, will be necessary to validate the proposed approach.

## 6. Patents

An Italian Patent application (102022000021093) has been submitted regarding this experimental work.

## Figures and Tables

**Table 1:** Characterization of Mango and Shea SLNs by hot and cold saponification. % recovery, EE% of Bume and Dexa. % recovery, EE% of 6-cou, rhod B-AOT, and rhod B. Abbreviations: Bume: bumetanide; Dexa: dexamethasone; 6-cou: 6-coumarin; C_3_H_6_O_3_: lactic acid; H_3_PO_4_: phosphoric acid; rhod B: rhodamine B; AOT: docusate sodium salt; SLNs: solid lipid nanoparticles.

	Saponification		Precipitation Acid	Soap	Mean Particle Size (nm)	Polydispersion	% Recovery	EE%
	Cold	Hot						
Mango SLNs	x		H_3_PO_4_	1%	259.1 ± 2.5	0.179	-	-
Shea SLNs					299.5 ± 1.7	0.173	-	-
Shea SLNs		x		4%	120.4 ± 1.0	0.138	-	-
Mango SLNs		x		1%	178.9 ± 0.8	0.202	-	-
Shea SLNs					131.1 ± 1.2	0.197	-	-
Mango SLNs		x		1%	191.8 ± 1.9	0.081	6-cou 79	6-cou 93
Shea SLNs					143.1 ± 3.4	0.056	6-cou 95	6-cou 92
Mango SLNs		x		1%	360.5 ± 2.6	0.110	rhod B-AOT 77	rhod B-AOT 86
Shea SLNs					298.7 ± 1.6	0.173	rhod B-AOT 89	rhod B-AOT 85
Mango SLNs		x		1%	323.6 ± 5.0	0.106	rhod B 64	rhod B 38
Shea SLNs					443.9 ± 4.0	0.084	rhod B 72	rhod B 55
Mango SLNs		x	C_3_H_6_O_3_	1%	228.5 ± 0.7	0.226	Bume 72 ± 6.13	Bume 74 ± 3.3
							Dexa73 ± 11.98	Dexa 25 ± 8.33
Shea SLNs					201.1 ± 1.4	0.108	Bume 70 ± 6.05	Bume 83 ± 1.63
							Dexa 80 ± 4.33	Dexa 53 ± 12.93

**Table 2:** Measures of enthalpy, T_onset_, and T_peak_ for Mango and Shea solid lipid nanoparticles (SLNs), fatty acids from Shea and Mango soaps, and synthetic fatty acids.

	T_onset_	T_peak_	Enthalpy (J/g)
Mango SLNs	54.06	59.61	21.29
Mango fatty acids	56.66	59.61	11.38
Shea SLNs	44.72	53.81	17.28
Shea fatty acids	45.69	52.7	22.98
Palmitic acid	59.64	63.59	195.23
Stearic acid	68.06	69.17	188.61
Stearic acid by coacervation	51.8	54.71	165.43
Arachidic acid	70.71	71.83	202.94

**Table 3:** Lipid composition and percentage in the obtained green SLN pellet, subjected to derivatization. Results are expressed as a percentage in the dry pellet. ^a^ Linear retention index; ^b^ [39,40]; ^c^ all compounds were confirmed by co-injection of the authentic reference standard.

		*I* ^t a^				Cold Saponification		Hot Saponification	
Peak N°	Retention Time	Experimental	Reference ^b^	Compound Name ^c^	MW (TMS)	Mango Pellet	Shea Pellet	Mango Pellet	Shea Pellet
1	23.864	1288	1290	Glycerol 3TMS	308	0.73% (rsd%:12.7%)	0.77% (rsd%:3.7%)	0.83% (rsd%:17.4%)	1.11% (rsd%:19.9%)
2	52.905	2054	2053	Palmitic acid TMS	328	6.84% (rsd%:10.8%)	3.31% (rsd%:10.5%)	2.83% (rsd%:33.2%)	3.84% (rsd%:11.7%)
3	57.928	2217	2217	Linoleic acid TMS	352	3.49% (rsd%:18.3%)	3.94% (rsd%:1.2%)	0.37% (rsd%:39%)	2.54% (rsd%:25.6%)
4	58.076	2222	2224	Oleic acid TMS	354	35.99% (rsd%:12.2%)	36.81% (rsd%:8.3%)	30.84% (rsd%:21%)	47.55% (rsd%:14.1%)
5	58.880	2249	2250	Stearic acid TMS	356	24.87% (rsd%:11.4%)	25.68% (rsd%:10.7%)	24.69% (rsd%:27.3%)	26.98% (rsd%:3.6%)
6	64.511	2449	2451	Arachidic acid TMS	384	0.97% (rsd%:8.1%)	0.91% (rsd%:18.7%)	2.65% (rsd%:9.4%)	0.84% (rsd%:6.6%)
7	68.780	2611	2611	Glyceryl palmitate 2TMS	474	0.32% (rsd%:26.9%)	0.28% (rsd%:9.6%)	<0.25% (~0.12%) (rsd%:34.2%)	<0.25% (~0.24%) (rsd%:30.4%)
8	72.949	2779	2788	Glyceryl oleate 2TMS	500	5.24% (rsd%:18.7%)	7.30% (rsd%:12.8%)	1.59% (rsd%:36.3%)	3.52% (rsd%:22.8%)
9	73.540	2803	2806	Glyceryl stearate TMS	502	4.61% (rsd%:17%)	6.48% (rsd%:18.6%)	1.61% (rsd%:37.3%)	3.48% (rsd%:19.8%)

**Table 4:** List of identified and hypothesized compounds in Mango and Shea soap and in the relative SLNs. For each compound, the relative retention time, the maximum UV absorption, the protonated and deprotonated molecules, and the names of the compounds are given. ^a^ Compound confirmed by co-injection of the authentic reference standard; ^b^ see Section 2.9.2; semiquantitative results expressed as * cinnamic acid; ** conjugated linolenic acid; *** lupeol cinnamate; and n.d., not detected.

								Quantification as µg/mg Soap (RSD%)		PA_p_/(PA_p_ + PA_s_) ^b^	
Peak N°	Retention Time	UV	ESI^+^	ESI^−^	MW	Compound	Reference	Mango	Shea	Mango	Shea
1	28.043	277	190 [M+H+CH_3_CN]^+^	147 [M-H-]^−^, 193 [M-H+HCOOH]^−^	148	Cinnamic acid ^a^*	[49,50]	0.28 (8.4%)	0.42 (5.7%)	0.25	0.20
2	45.825	224/270/ 261/281	279 [M+H]^+^	/	278	Conjugated trienoic fatty acid derivatives **	[51]	0.09% (17.4%)	0.15% (9.7%)	1	1
3	73.614	223/273	409 [M+H-C_9_H_8_O_2_]^+^/ 557 [M+H]^+^	/	556	Lupeol Cinnamate ^a^***	[26,47]	2.21 (17%)	0.72 (14.6%)	0.70	0.62
4	75.955	223/273	409 [M+H-C_9_H_8_O_2_]^+^	/	/	Triterpene cinnamyl ester derivative ***	[26,47]	2.98 (17%)	1.01 (14.6%)	1	1
5	80.862	224/272	409 [M+H-C_9_H_8_O_2_]^+^	/	/	Triterpene cinnamyl ester derivative ***	[26,47]	1.24 (16.6%)	0.41 (17.8%)	1	1
6	83.964	224/273	409 [M+H-C_9_H_8_O_2_-H_2_O]^+^	/	/	Triterpene cinnamyl ester derivative ***	[26,47]	1.64 (12.8%)	0.52 (11.3%)	1	n.d.

## Data Availability

The data presented in this study are available on request from the corresponding author.

## Supplementary Materials

The following supporting information can be downloaded at https://www.mdpi.com/article/10.3390/pharmaceutics16081051/s1, Table S1. Target ion, qualifier ions, linearity range, R2, and calibration curve of derivatized compounds quantified by GC-MS. Figure S1. DSC thermograms. (A) Green: palmitic acid; red: stearic acid; black: stearic acid by coacervation; blue: Shea fatty acids; pink: Mango fatty acids. (B) Green: Shea solid lipid nanoparticles (SLNs); red: Shea fatty acids; black: Mango SLNs; blue: Mango fatty acids. Figure S2. Optical microscopy of Shea and Mango SLNs loaded with Bume-Dexa and without loaded drugs. Figure S3. Optical microscopy of Shea and Mango SLNs labelled with 6-Cou and the Rhod B-AOT ion pair. Abbreviations: 6-cou: 6-coumarin; rhod B: rhodamine B. Table S2. Particle size of Mango and Shea SLNs by hot saponification after 30 days of storage at 4 °C. Abbreviations: Bume: bumetanide; Dexa: dexamethasone; 6-cou: 6-coumarin; C_3_H_6_O_3_: lactic acid; H_3_PO_4_: phosphoric acid; rhod B: rhodamine B; AOT: docusate sodium salt; SLNs: solid lipid nanoparticles. Figure S4. (A) GC-MS profiles of Shea soap as such and its SLN pellet after derivatization. The profiles are compared with the blank SLNs (derivatization performed without the lipid matrix). Peak numbers refer to Table 3. (B) GC-MS profiles of Mango soap as such and its SLN pellet after derivatization. The profiles are compared with the blank SLNs (derivatization performed without the lipid matrix). Peak numbers refer to Table 3. * Phosphate TMS. Figure S5. (A) UHPLC-PDA profiles (λ = 270 nm) of Shea soap as such and its SLNs, split into pellet and supernatant. The profiles are compared with the blank SLNs (extraction without matrix). Peak numbers refer to Table 4. (B) UHPLC-PDA profiles (λ = 270 nm) of Mango soap as such and its SLNs, split into pellet and supernatant. The profiles are compared with the blank SLNs. Peak numbers refer to Table 4. Figure S6. Sheep CPEC fluid secretion assay with the incubation buffer in the apical chamber.
