# Supplementary material for: Green Solid Lipid Nanoparticles by Fatty Acid Coacervation: An Innovative Nasal Delivery Tool for Drugs Targeting Cerebrovascular and Neurological Diseases
Source: Pharmaceutics. 2024 Aug 8;16(8):1051. doi: 10.3390/pharmaceutics16081051 (PMC11360092; doi:10.3390/pharmaceutics16081051)
Supplement: Supplementary file 1 [file pharmaceutics-16-01051-s001.zip › pharmaceutics-3112014-supplementary.pdf]

“Green” Solid Lipid Nanoparticles (SLNs) by fatty acid coacervation: an innovative nasal delivery tool for drugs targeting cerebrovascular and neurological diseases

Annalisa Bozza, Valentina Bordano, Arianna Marengo, Elisabetta Muntoni, Elisabetta Marini, Loretta Lazzarato, Chiara Dianzani, Chiara Monge, Arianna Carolina Rosa; Luigi Cangemi; Maria Carmen Valsania, Barbara Colitti, Ezio Camisassa and Luigi Battaglia

Supplementary materials

**Table S1**	**page 2**
**Figure S1**	**page 3**
**Figure S2**	**page 4**
**Figure S3**	**page 5**
**Table S2**	**page 6**
**Figure S4**	**page 7**
**Figure S5**	**page 8**
**Figure S6**	**page 9**

Table S1. Target ion, qualifier ions, linearity range, R^2, and calibration curve of derivatized compounds quantified by GC-MS.

Compound Name	Target Ion	Qualifier Ions	Linearity Range (µg/mL)	R^2	Calibration Curve
Glycerol 3TMS	147	117,205	100-1000	0.9847	y = 6683.6x - 101540
Palmitic acid TMS	313	132,328	100-1000	0.9968	y = 2058.8x - 41546
Linoleic acid TMS	337	129,262	100-1000	0.9991	y = 475.48x - 10931
Oleic acid TMS	339	129, 354	100-1000	0.997	y = 452.98x - 9579
Stearic acid TMS	341	132,356	100-1000	0.9984	y = 1758.9x - 42104
Arachidic acid TMS	369	117,145	100-1000	0.9996	y = 1076.7x - 23013
Glyceryl palmitate 2TMS	371	239,459	25-100	0.9791	y = 2022.8x - 9379.5
Glyceryl oleate 2TMS	129	129,203	100-1000	0.982	y = 708.85x - 23322
Glyceryl stearate TMS	399	205,267	100-1000	0.9727	y = 968.71x - 32283

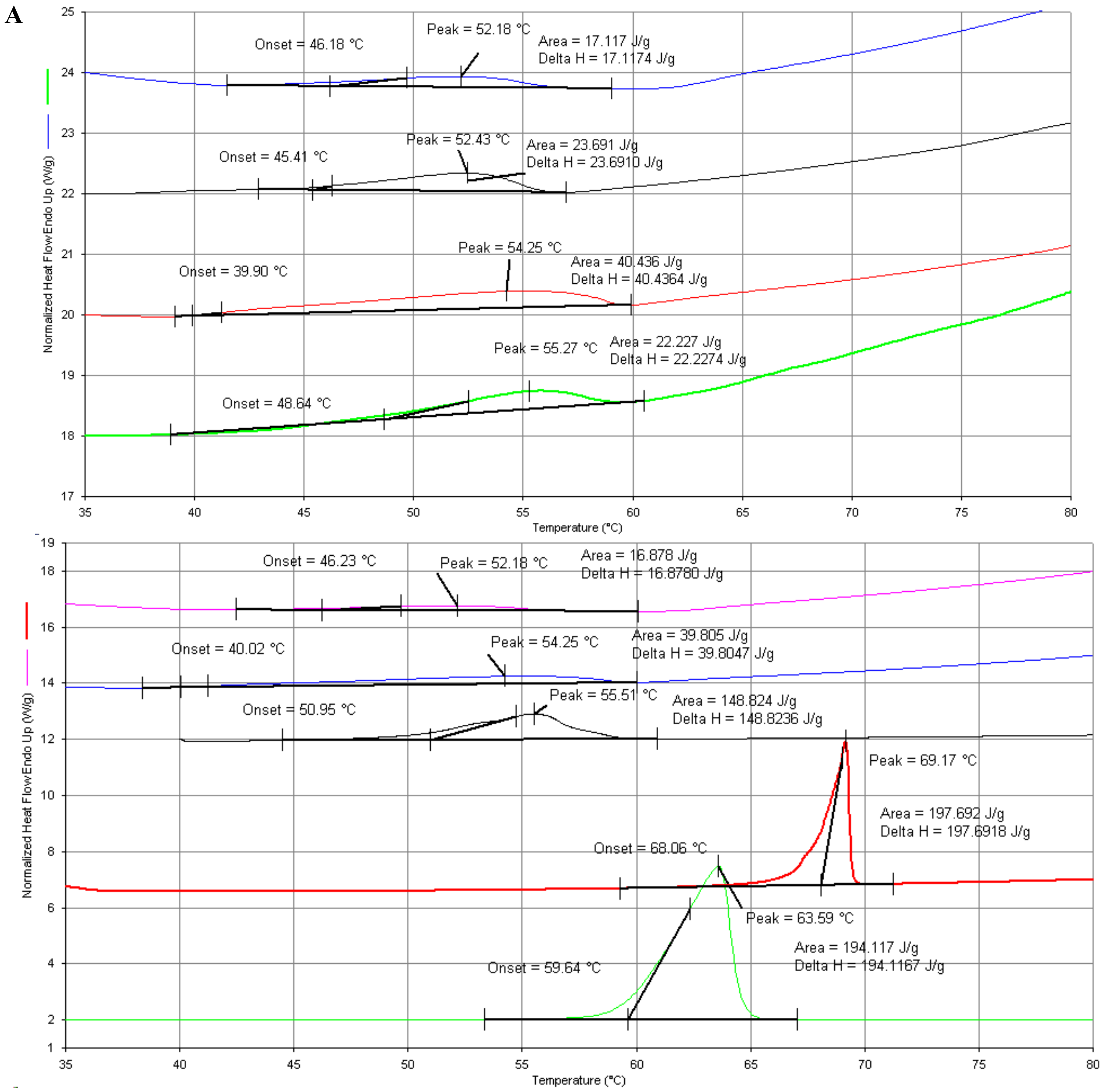

Figure S1. DSC thermograms. (A) Green: palmitic acid; red: stearic acid; black: stearic acid by coacervation; blue: Shea fatty acids; pink: Mango fatty acids. (B) Green: Shea solid lipid nanoparticles (SLNs); red: Shea fatty acids; black: Mango SLNs; blue: Mango fatty acids.

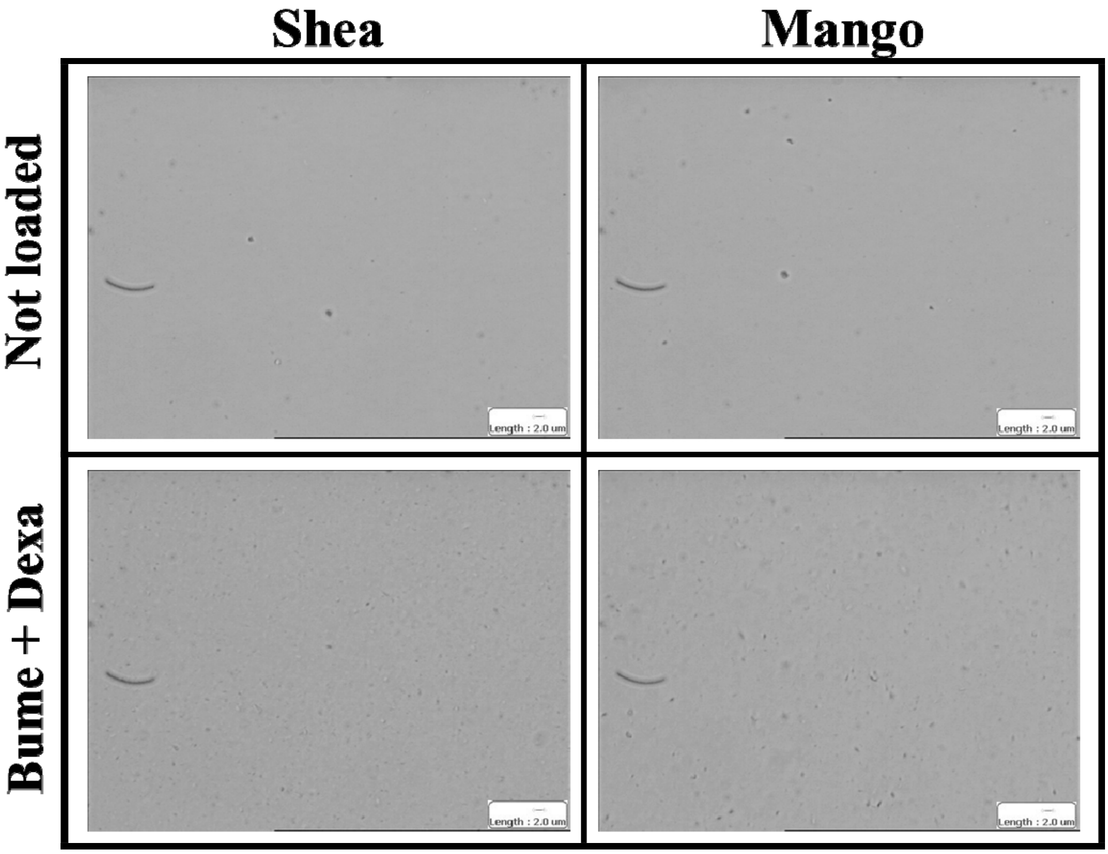

Figure S2. Optical microscopy of Shea and Mango SLNs loaded with Bume-Dexa and without loaded drugs.

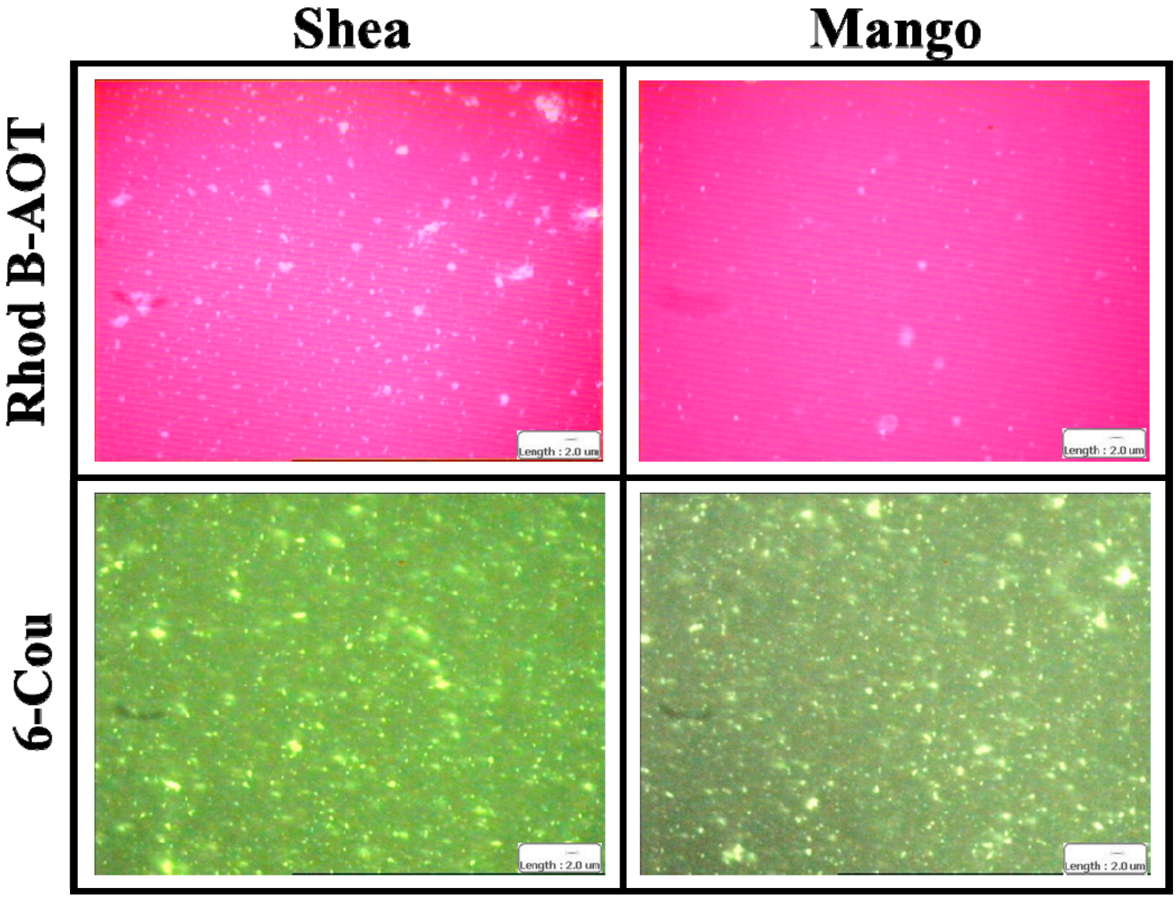

Figure S3. Optical microscopy of Shea and Mango SLNs labelled with 6-Cou and the Rhod B-AOT ion pair. Abbreviations: 6-cou: 6-coumarin; rhod B: rhodamine B.

Table S2. Particle size of Mango and Shea SLNs by hot saponification after 30 days of storage at 4 °C. Abbreviations: Bume: bumetanide; Dexa: dexamethasone; 6-cou: 6-coumarin; $C_3H_6O_3$: lactic acid; H_3PO_4: phosphoric acid; rhod B: rhodamine B; AOT: docusate sodium salt; SLNs: solid lipid nanoparticles.

T = 30 days at 4 °C							
	Saponification		Precipitation acid	Soap	Mean particle size (nm)	Polydispersion	Loaded compounds
	Cold	Hot					
Mango SLNs		x	H_3PO_4	1%	343.6 ± 33.3	0.133	-
Shea SLNs					437.0 ± 11.7	0.143	
Mango SLNs		x		1%	214.2 ± 1.1	0.138	6-cou
Shea SLNs					147.6 ± 4.7	0.087	
Mango SLNs		x		1%	372.8 ± 6.8	0.034	rhod B-AOT
Shea SLNs					498.1 ± 4.2	0.068	
Mango SLNs		x		1%	414.4 ± 7.7	0.158	rhod B
Shea SLNs					508.9 ± 6.5	0.148	
Mango SLNs		x	$C_3H_6O_3$	1%	443.1 ± 3.0	0.161	Bume and Dexa
Shea SLNs					403.3 ± 2.9	0.270	

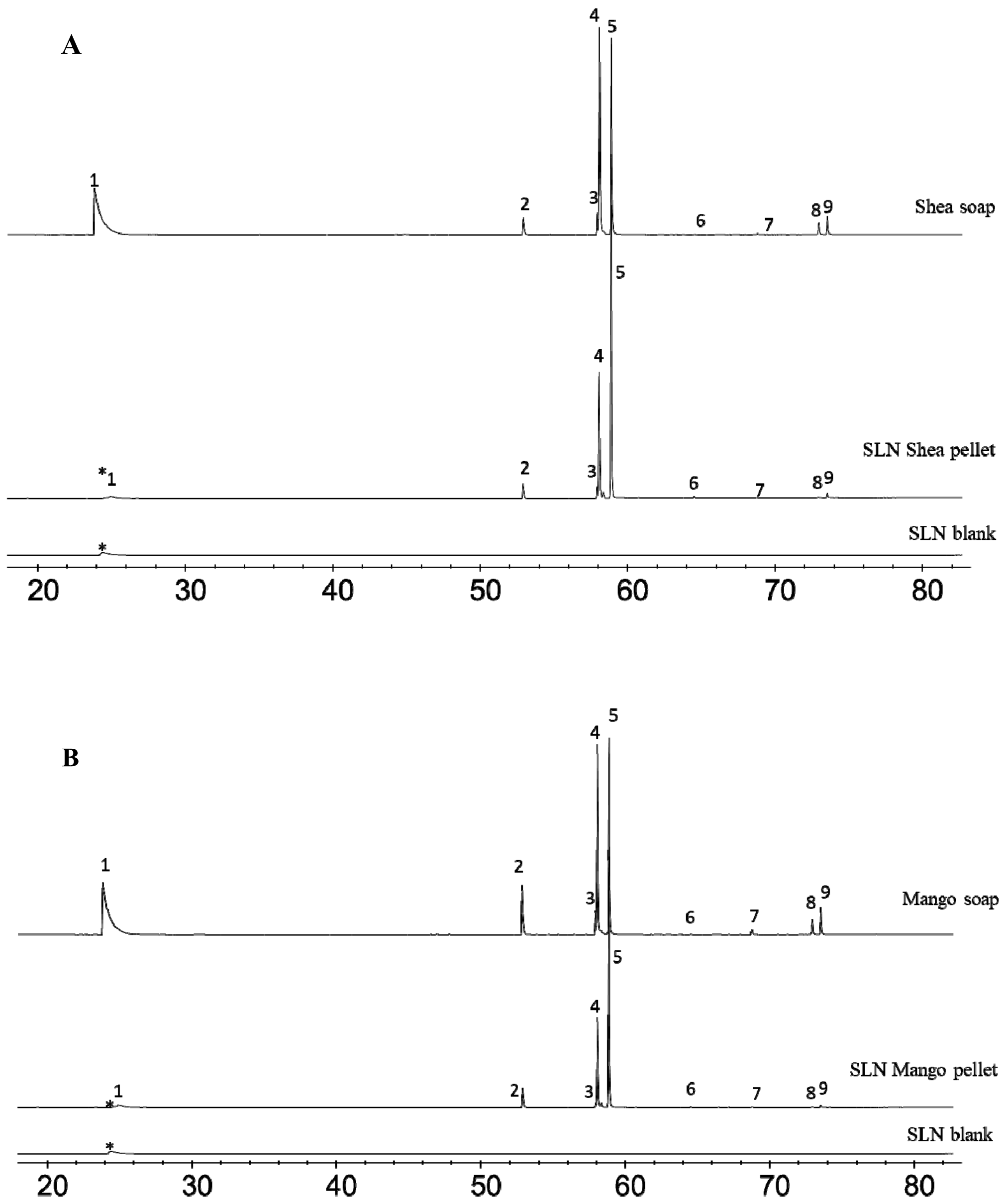

Figure S4. (A) GC-MS profiles of Shea soap as such and its SLN pellet after derivatization. The profiles are compared with the blank SLNs (derivatization performed without the lipid matrix). Peak numbers refer to Table 3. (B) GC-MS profiles of Mango soap as such and its SLN pellet after derivatization. The profiles are compared with the blank SLNs (derivatization performed without the lipid matrix). Peak numbers refer to Table 3. *Phosphate TMS.

A

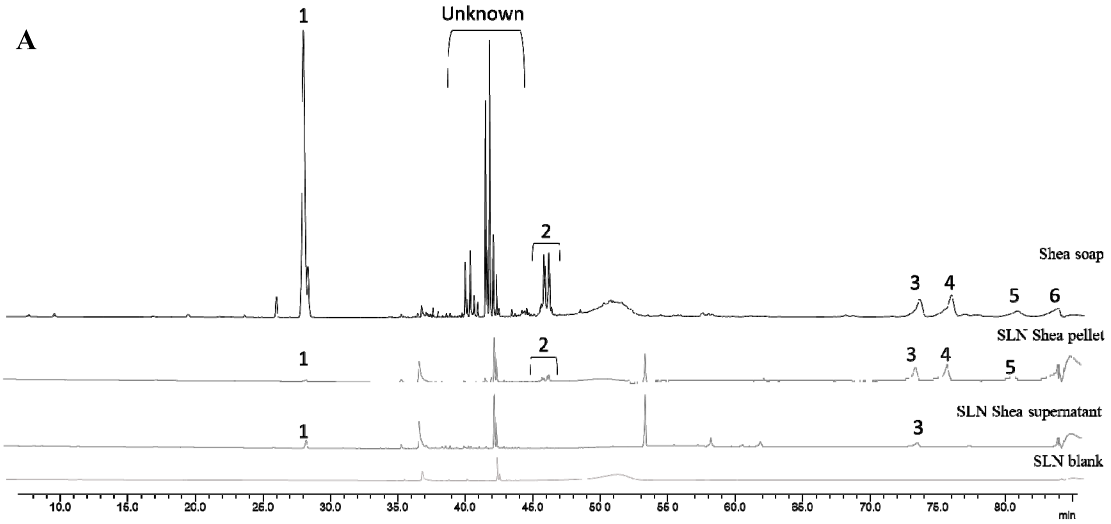

B

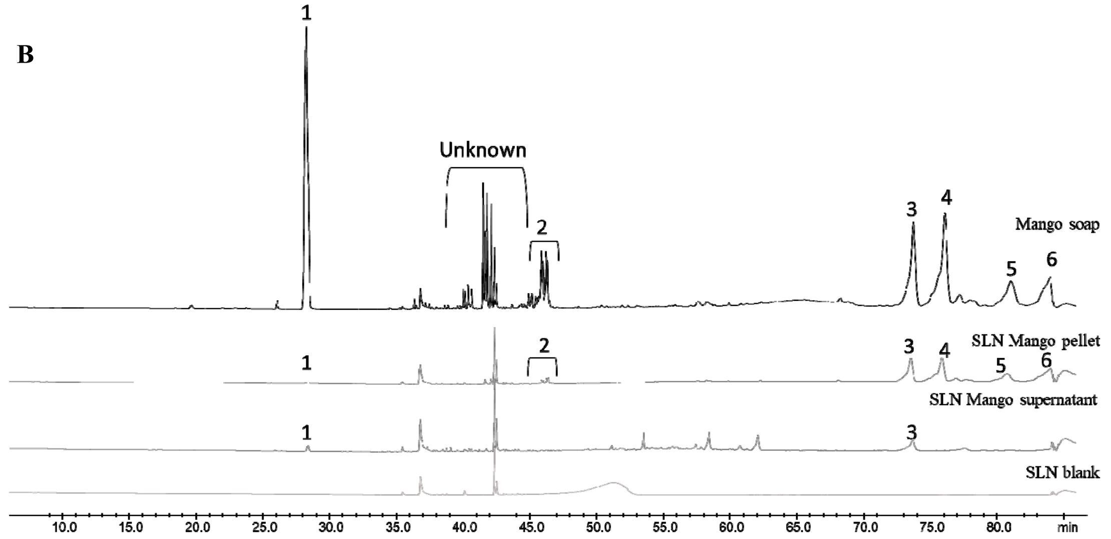

Figure S5. (A) UHPLC-PDA profiles (λ=270 nm) of Shea soap as such and its SLNs, split into pellet and supernatant. The profiles are compared with the blank SLNs (extraction without matrix). Peak numbers refer to Table 4. (B) UHPLC-PDA profiles (λ=270 nm) of Mango soap as such and its SLNs, split into pellet and supernatant. The profiles are compared with the blank SLNs. Peak numbers refer to Table 4.

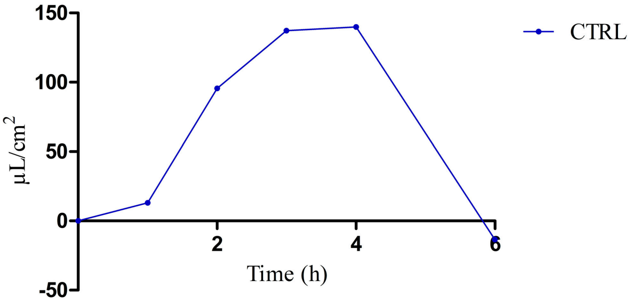

Figure S6. Sheep CPEC fluid secretion assay with the incubation buffer in the apical chamber.
